# The SNARC Effect in Chinese Numerals: Do Visual Properties of Characters and Hand Signs Influence Number Processing?

**DOI:** 10.1371/journal.pone.0163897

**Published:** 2016-09-29

**Authors:** Karl K. Kopiske, Christian Löwenkamp, Owino Eloka, Florian Schiller, Chung-Shan Kao, Chaohua Wu, Xiaorong Gao, Volker H. Franz

**Affiliations:** 1 Istituto Italiano di Tecnologia (IIT), Center for Neuroscience and Cognitive Systems@UniTn, Corso Bettini, 31, 38068 Rovereto, TN, Italy; 2 University of Hamburg, Institute of Psychology, Von-Melle-Park 5, 20146 Hamburg, Germany; 3 Justus Liebig University Giessen, Department of General Psychology, Otto-Behaghel-Straße 10, 35394 Giessen, Germany; 4 Tsinghua University, Department of Biomedical Engineering, Beijing 100084, PR China; 5 University of Tübingen, Institute of Computer Science, Department of Experimental Cognitive Science, Sand 13, 72076 Tübingen, Germany; Katholieke Universiteit Leuven, BELGIUM

## Abstract

The SNARC effect refers to an association of numbers and spatial properties of responses that is commonly thought to be amodal and independent of stimulus notation. We tested for a horizontal SNARC effect using Arabic digits, simple-form Chinese characters and Chinese hand signs in participants from Mainland China. We found a horizontal SNARC effect in all notations. This is the first time that a horizontal SNARC effect has been demonstrated in Chinese characters and Chinese hand signs. We tested for the SNARC effect in two experiments (parity judgement and magnitude judgement). The parity judgement task yielded clear, consistent SNARC effects in all notations, whereas results were more mixed in magnitude judgement. Both Chinese characters and Chinese hand signs are represented non-symbolically for low numbers and symbolically for higher numbers, allowing us to contrast within the same notation the effects of heavily learned non-symbolic vs. symbolic representation on the processing of numbers. In addition to finding a horizontal SNARC effect, we also found a robust numerical distance effect in all notations. This is particularly interesting as it persisted when participants reported using purely visual features to solve the task, thereby suggesting that numbers were processed semantically even when the task could be solved without the semantic information.

## 1 Introduction

### 1.1 The SNARC effect: Description and models

The SNARC (**S**patial **N**umerical **A**ssociation of **R**esponse **C**odes) effect refers to a proposed association between numbers and space. This was originally put forward by Dehaene, Bossini and Giraux [1], who found that European participants responded quicker to relatively smaller numbers with a response button on the left than on the right, and vice-versa for relatively large numbers, despite number magnitude being irrelevant for the task. This association is thought to be automatic [2]. The SNARC effect is found in a number of tasks (e.g., judgement of parity [1] or magnitude [3], but even unrelated tasks like finger tapping [4] have been shown to elicit SNARC-like effects). The most common task is a parity judgement task with horizontally separated response buttons: Participants are asked if a number is odd or even, and respond by pressing one of two buttons either on the left or on the right. The mapping of odd and even to buttons on the left and on the right will change during the experiment, so that for each number, left and right responses are given. The variable of interest then is the difference between right-handed and left-handed response times for each number. Statistically speaking, this is sometimes referred to as difference of response times, or dRT [5], where a systematic linear relationship between number magnitude and dRT indicates a SNARC effect.

Several models have been put forward to explain the effect. The most prominent explanation has been the following: during number processing, an internal mental number line is automatically activated. In Western participants, number magnitude will ascend from left to right on this line so that small numbers will be on the left and large numbers on the right [6–9]. The mental number line is mapped into external space; the better mapping location and response location correspond, the faster the response, giving rise to the SNARC effect. In Western participants, for example, small numbers will be mapped to the left of the number line and larger numbers to the right of the number line. This has also been called the direct mapping account [9], since it suggests a direct relationship between numbers and space. However, competing accounts exist. For example, the dual-route explanation [10,11] proposes that the activation of the mental number line is not necessary in numerical cognition and that humans may manipulate numbers without accessing their semantic meaning, purely through automatic activation of a response associated with the word or symbol. According to the dual-route explanation, the SNARC effect can also arise, but only when it is necessary to extract the meaning of a number word, which would then activate the number line. A more general approach than that of a mental number line was put forward by Proctor and Cho [12,13]. They assume that each number and response to some extent has a positive or negative polarity, and that the SNARC is a congruency effect of polarities. For a summary of the ongoing debate about which model best describes the SNARC effect and related findings, see van Dijk, Gevers, Lafosse and Fias [14].

### 1.2 To what degree does the SNARC effect depend on notation?

A large number of notations and modalities have been found to elicit a SNARC effect or SNARC-like effects. These include, besides Arabic digits, visual and auditory number words, dice patterns [15], letters of the alphabet, months [16], learned magnitude relations [17], German Sign Language hand signs [18], as well as Japanese [19] and Chinese characters, although the strength and direction of a SNARC effect may differ [20]. Thus, it is often claimed that the SNARC effect is amodal and independent of notation [15,21]. However, studies have reported results that the direction of number-space mapping depends on interpretation of the numbers [22], typical context of a symbol [20] or even single number words in a language written in a different direction presented in a previous trial [23], which has prompted others to say that the effect may be more dependent on the particular stimuli and experimental design used. For reviews on this issue, see Gevers and Lammertyn [24] or Wood, Willmes, Nuerk and Fischer [25].

Indeed, where a SNARC effect can and cannot be found has informed several hypotheses about underlying mechanisms of the effect. One example is the common notion that the effect is strongly influenced by reading habit (see e.g. [24,26]). This was already hypothesised in the original paper [1], which reported a strong left to right SNARC effect in French participants, but a significantly weaker effect in Iranians (whose native language is written right-to-left) living in France, with the strength of the SNARC effect possibly related to the length of time since moving to France. This influence is thought to hold for both reading habit of numbers and of words, as Shaki, Fischer and Petrusic [27] found by testing Israeli (reading habit for words: right to left, numbers: left to right; no clear SNARC effect) and Palestinian participants (words and numbers: right to left; clear right to left SNARC effect). Similar findings have supported this idea, as a vertical SNARC effect (speed-advantage of top-small and large-bottom responses over top-large and small-bottom) has been demonstrated in Taiwanese participants [20], who may read a significant portion of text vertically. A vertical SNARC effect was also demonstrated in Japanese participants [19], although the direction was reversed here, with smaller numbers being responded to faster when the response was on the lower of two vertically separated response buttons. This is interesting since reading habit would be the same in the samples studied by Hung et al. [20] and Ito and Hatta [19], and indeed Ito and Hatta do not cite reading habit as an explanation for their vertical effect at all, but rather explain it in terms of a separate, general association of magnitude and space [19].

This idea of a general magnitude-space association is supported by findings that a vertical SNARC effect can also be found in European and American participants [28–30], although it appears to be less automatically activated than the horizontal SNARC in tasks where the magnitude of the stimulus or the vertical dimension of the response is irrelevant [28,29]. Such associations in a direction that does not correspond with typical reading habit are central evidence for the notion of a general magnitude-space association, although they have also been taken as evidence that the effect may be more dependent on short-term influences as opposed to long-term habits than previously assumed [23].

These associations are also very relevant to the question of which mechanisms might be behind a general association of space and magnitude. In addition to the polarity account of Proctor and Cho [12], it has also been related to grounded cognition by Fischer and Brugger [31], who proposed three levels of stimulus-dependent space-magnitude associations: (a) Grounded representations, in which basic properties of the world determine the associations (e.g., the fact that stacking objects creates increasingly higher piles would contribute to a large-upwards association) (b) embodied cognition, where representations are influenced by associations with body parts, such as hands or fingers (c) situated cognition, with the representation being dependent on the context. Evidence for this view has come for example from Bächtold et al. [22], where conceptualising numbers as a ruler or a clock produced opposite results. Additionally, finger counting habit has also been found to affect the SNARC effect (e.g. [32]; see [33] for a review and [34] for an imaging study reporting consistent modulations of neural activation), in that the strength of the SNARC effect differed between participants that start counting on their left hand and those that start on their right hand. Within such a framework [31], studying effects of numerical cognition and specifically the SNARC effect in hand signs is especially interesting, as the notion of embodied cognition would predict properties of these hand signs to directly influence the spatial representation of magnitude presented through hand signs.

### 1.3 Our study: Employing Chinese characters to investigate notation dependence

Our study tested whether or not a horizontal SNARC effect can be found for *Arabic digits*, simple-form *Chinese characters*, and *Chinese hand signs* in participants living and raised in Mainland China. The *Arabic digits* notation was included to measure the well-known horizontal SNARC effect as a baseline to compare the other notations to, while each of the other notations allowed us to test specific predictions.

It has been proposed that generally, number processing in Chinese speakers may differ fundamentally from number processing in Western participants [35]. It has also been suggested that the semantic processing of Chinese characters differs from that of *Arabic digits* [35,36], specifically with regard to its temporal properties. More specifically, while Mainland Chinese and Taiwanese participants display a horizontal SNARC effect in Arabic digits [20,37], Taiwanese participants may not display a clear horizontal SNARC effect for *Chinese characters* [20]. The main difference between participants from Taiwan and Mainland China is that whereas horizontal writing was formally introduced in mainland China in 1955 and used henceforth, in Taiwan a similar guideline was introduced for official documents in 2004 (see [38], accessed via [39]), and there remain texts (including e.g. books, textbooks) that are written vertically. Thus, the exposure to vertical text would be greatly different, despite the characters being the same. Finding a clear horizontal SNARC effect for *Chinese characters* in our Mainland Chinese participants would emphasise the importance of reading and writing experience and provide more evidence for it being independent of notation, while the opposite finding would point towards there being an effect of notation.

**Fig 1:**
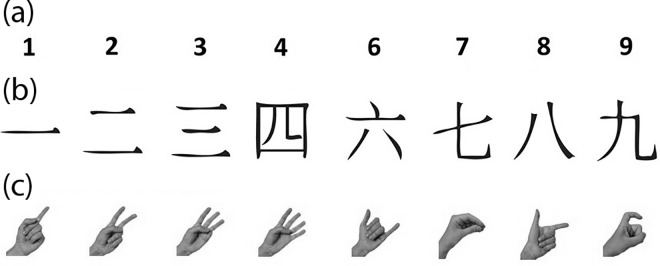
The stimuli used in our experiments. (a) Arabic digits, (b) simple-form Chinese characters, (c) Chinese hand signs as used in Chinese Sign Language. Stimuli in each column represent identical numbers. Note that the number 5 is omitted in all notations. This enabled us to use it as the standard for the magnitude judgement task. Hand signs images retrieved from https://en.wikipedia.org/wiki/Chinese_number_gestures, created by Wikipedia user Ningling, and used under the terms of the GNU Free Documentation License.

*Chinese hand signs* were included to test for the effect in a neither purely symbolic, nor purely non-symbolic notation in which the effect had never been tested before. It has been shown that notations defined by numerosity (e.g. dice patterns, [15]) can evoke a SNARC effect, as can hand signs[18,40]. However, in Western sign languages, numbers tend to be represented by the same number of fingers, making it a non-symbolic representation based on finger numerosity. Chinese hand signs, on the other hand, are represented through finger numerosity for numbers 1…5, and purely symbolically for numbers 6…10 (displayed through different signs using one, two, or three fingers–see Fig 1), which means that one cannot assume that the same effect will necessarily be present. Finding a SNARC effect in this notation, and especially in the higher (symbolically represented) range, would indicate that indeed the magnitude displayed in hand signs can elicit the effect independently of the number of fingers seen. Such a finding would also make this notation potentially a beneficial, confound-free embodied notation to test predictions of embodied cognition with regards to participant counting preferences or body postures. On the other hand, a difference between symbolically and non-symbolically represented numbers would reinforce the notion that hand signs where the number of fingers represents magnitude are susceptible to a confound of magnitude and numerosity.

### 1.4 Our study: Methodology and research questions

We tested the effect in the two most common tasks in the SNARC literature: Parity judgement (i.e., judging if a number is odd or even) and magnitude judgement (i.e., judging if a number is smaller or larger than a standard, in this case 5). Beside the obvious difference between the tasks, they may also differ with regards to the information that is activated, as previous studies have shown that secondary tasks that tax working memory suppress the SNARC effect in *parity* judgement when *verbal* resources are required for the secondary task, whereas the SNARC effect in *magnitude* judgement is suppressed by *visuospatial* secondary tasks [3,41]. If parity judgement and magnitude judgement depend primarily on verbal and visuospatial information, respectively, it would be plausible for magnitude judgement to be more affected by the differences in visual complexity between the notations. We also tested for a numeric distance effect in the magnitude judgement task (i.e., faster response times when the numerical distance between the stimulus and the standard is larger), a common marker of semantic processing [7,42], to test whether the magnitude judgement task was indeed executed based not on purely visual features but based on magnitude information, which would be a prerequisite to interpret the results from it as indicative of a space-number association.

In summary, we tested for the existence of a horizontal SNARC effect in native Chinese participants living in Beijing using three different notations and two tasks. These notations and tasks allowed us to (a) investigate the processing of a mixed symbolic and non-symbolic (numerosity-based), embodied notation (b) separate reading habit and notation for Chinese characters by comparing our results to those of a study conducted with Taiwanese participants, and (c) test if our results would persist under different task demands inherent to parity judgement and magnitude judgement.

## 2 Experiment 1: Parity judgement

A classic parity judgement paradigm was used in experiment 1, in the three different notations *Arabic numbers*, *Chinese characters*, and *Chinese hand signs*. The goal was to test for the existence of a horizontal SNARC effect in native Chinese speakers who grew up in Mainland China, using the most common task for SNARC experiments.

### 2.1 Participants

Twenty-six native Chinese speakers (all at least 18 years old, age: M = 22.5, SD = 2.0), recruited September 2011, participated voluntarily for an agreed pay of 50 RMB. All were right-handed by self-report, 12 were female. All had normal or corrected-to-normal vision. All were students of Tsinghua University, Beijing, and naive to the purpose of the task.

All participants were adults, 18 years or older, recruited from the Tsinghua University Biomedical Engineering department. Participants gave written, informed consent. They were compensated by a previously agreed amount (see specifics for each experiment in the manuscript) that reflected the standard payment in the department. Participants signed their name on the consent sheet, but no identifying information was contained in the experimental data itself, and the consent sheet could not be linked to any data. On the consent sheet, participants also confirmed that they had normal or corrected-to-normal vision and hearing, as well as no attention disorders. Participants that did not meet all of these criteria were not tested, and no data or records of any kind were recorded of them. The information was also not linked to any information in the data, and no further medical information was collected. Consent sheets are being stored in a locked cabinet at the University of Hamburg Psychology department that is only accessible to members of the department.

Both experiments were conducted in accordance with to the 1964 Declaration of Helsinki, and following ethical guidelines of the German Psychological Society (DGPs) and the Professional Association of German Psychologists (BDP) (2005, C.III). The study was conducted within the International Graduate Research Group "Cross-modal Interaction in Natural and Artificial Cognitive Systems" (CINACS) that was reviewed and approved by the German Research Foundation (DFG, project number IGK-1247) which did not require further Institutional Review Board approval. The reviewed description of this research group included response time tasks like the ones conducted for this study.

Our experiments lasted at most 65 minutes, during which participants were allowed to take as many breaks as they wished. They consisted of standard two-alternative-forced-choice response time tasks that required quick button presses in response to numbers displayed on a standard computer screen. For these experiments, no particular risk of harm or stress was apparent to us, other than the possibility that participants may find the monotonous task somewhat tedious. Thus, we did not seek further ethical approval for this particular study. We retroactively asked the Local Ethics Committee of the Faculty for Psychology and Human Movement Science, University of Hamburg to assess whether ethical review would have been necessary. The committee came to the conclusion that this was not the case.

### 2.2 Stimuli and apparatus

Participants were seated approximately 60 cm in front of a 19” LCD monitor (effective screen diagonal: 48 cm) using a resolution of 1024 * 768 pixels with a refresh rate of 60 Hz and gave responses via a standard QWERTY USB-keyboard. They were presented with the numbers 1…4, 6…9 in three different notations: *Arabic numbers*, *Chinese characters*, and *Chinese number gestures* as used in Chinese Sign Language and displayed with the left hand, see Fig 1. Numbers were presented as 225 pixel * 225 pixel tagged image file (tif) images, centrally displaying Chinese and Arabic characters of font size 60 and images of hands of approximately equal size (app. 24 mm * 24 mm, corresponding to about 2.3 degrees of visual angle). The stimuli were presented in a custom MATLAB program using Psychophysics Toolbox 3 [43].

### 2.3 Procedure

The experiment was segmented into 6 blocks: Two blocks for each parity mapping, that is, *left = even; right = odd* and *left = odd; right = even*, for each of the three notations *Arabic digits*, *Chinese characters*, and *Chinese hand signs*. Each block consisted of practice trials until 8 correct responses were given, followed by 288 experimental trials (8 numbers * 36 repetitions). This resulted in at least 1776 trials total, and 1728 experimental trials. The order of blocks was counterbalanced between participants, with blocks of the same notation presented consecutively and each participant being assigned to one of 12 groups (6 sequences of blocks * 2 sequences of mapping). The numbers within each block were randomised. Before each block, participants were instructed which of the buttons “s”and “l”on the keyboard was to be pressed for even numbers and which for odd numbers. In each trial, a fixation cross (font size 40) appeared for a mean duration of 500 ms (400 ms + random value from an exponential distribution with mean = 100 ms), followed by the stimulus presented until a response button was pressed, but at most 2000 ms. This was followed by a pause of 250 ms. After every error, the participants saw the word “wrong!”written in red, font size 40, centrally on the screen for 250 ms. In total, the experiment lasted between 50 and 65 minutes.

### 2.4 Data analysis

A total of four participants had to be excluded from data-analysis: Two because of the number of errors made (more than 2 SD above the mean), one for being unfamiliar with some stimuli, and one because of technical difficulties. This left us with 22 participants for analysis. Response times were trimmed with 200 ms as the lower cut-off and each participant’s median RT for each notation plus three standard deviations as upper cut-offs, respectively, which eliminated 1.7% of trials from analysis.

We ran two main types of analysis: First, we ran a 3 (notation) * 2 (side of response) * 4 (numerical magnitude) * 6 (order) ANOVA over RTs. The factor *order* was a between-subject factor that coded the order of blocks. Numbers were assigned to four magnitude bins (1 and 2, 3 and 4, 6 and 7, 8 and 9) to control for confounding effects like the MARC effect (markedness association of response codes [44]) and to keep the analyses in line with recently proposed methodology [45,46]. Note that markedness, the property of being marked as unique, or uncommon [47], is a linguistic concept in which the English words “odd” and “even” differ, but not the Chinese equivalents. Thus, we did not expect the same mechanism to have an effect here. However, a similar effect would have been plausible: In Chinese, 奇偶, literally “odd even”, means parity. Hence, we tested for an advantage of “left-odd” and “right-even” responses. Greenhouse-Geisser-corrected p-values [48] along with Greenhouse-Geisser epsilon (ε_GG_) are given for factors with more than two levels. Second, we computed response time differences between right-handed responses and left-handed responses (dRTs) for each participant, number and notation, which we then used to compute linear regression slopes of dRT over number and magnitude bin (in *ms per digit* or *ms per bin*, respectively, see Table 1). For each notation, these slopes were then submitted to t-tests against 0 to clarify if the SNARC effect persisted in each notation. Third, we submitted these dRT slopes to a Bayesian analysis that can have some advantages over frequentist statistics (e.g., Dienes [49,50]) and allowed us to disambiguate whether non-significant results when testing for a SNARC effect, or for a difference between SNARC effects, should be interpreted as evidence for the absence of an effect or as a consequence of inconclusive data. We followed the guidelines proposed by Jeffreys [51] for the interpretation of Bayes factors (BFs). In short, throughout this paper we refer to BFs below 1/100 as decisive evidence for the H0, BFs below 1/10 as strong evidence for the H0, and BFs below 1/3 as substantial evidence for the H0. BFs between 1/3 and 3 indicate that the data are not sensitive enough to draw strong conclusions. BFs above 3, above 10, and above 100 represent substantial, strong, and decisive evidence in favour of the H1, respectively.

**Table 1:** Regression coefficients of dRT over number and dRT over magnitude bins.

*Notation*	*Regression dRT by number*	*R²*	*Regression dRT by magnitude bins*	*R²*
Digits exp. 1	y = 26.4–5.1x±0.9	.101	y = 31.3–12.2x±2.3	.096
Digits exp. 2	y = 28.9–7.5x±1.6	.246	y = 36.6–18.1x±3.9	.239
Characters exp. 1	y = 16.1–3.7x±1.1	.068	y = 20.2–9.0x±2.6	.067
Characters exp. 2	y = 12.8–4.5x±2.3	.080	y = 18.0–11.0x±5.4	.080
Hand signs exp. 1	y = 14.2–3.2x±1.1	.039	y = 15.7–7.0x±2.5	.032
Hand signs exp. 2	y = 7.8–2.6x±2.8	.021	y = 11.6–6.8x±6.8	.023

*Note*: Mean coefficients of the linear regression are given as y in ms, x in ms/digit and ms/bin, respectively, ± SEM. R² indicates explained variance. Experiment 1: Parity judgement, experiment 2: Magnitude judgement.

### 2.5 Results

**Fig 2:**
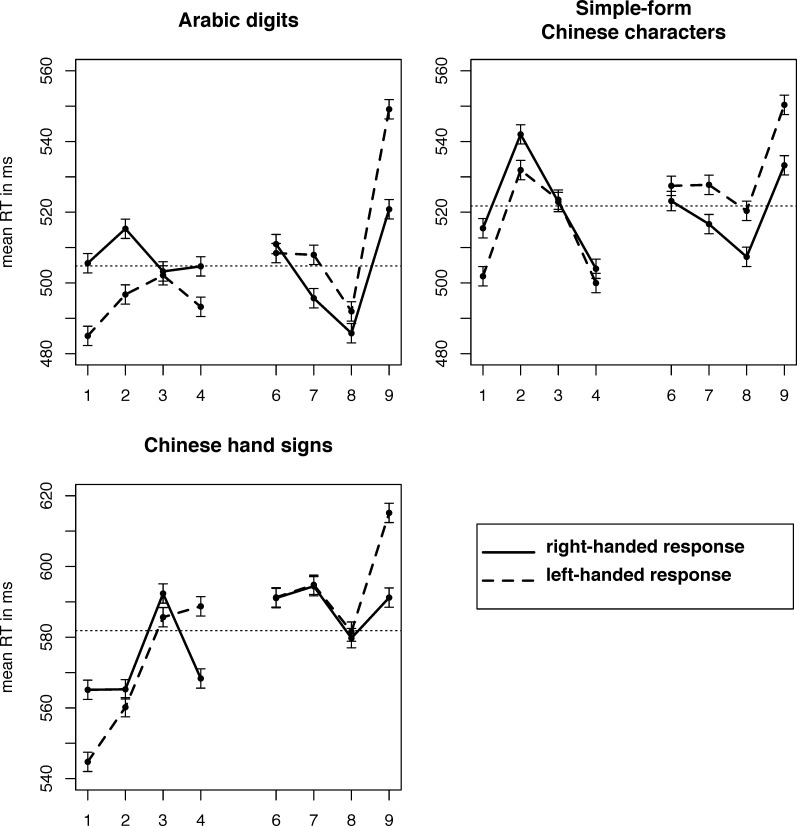
Left-handed and right-handed responses to each number in each notation, parity judgement task. **SNARC effect**: Right-handed responses slower than left-handed responses for small numbers, faster for large numbers. Error bars indicate within-subject SEMs for each number, pooled across each contrast of numbers [52,53]. Horizontal dashed lines indicate grand means of RTs for each notation.

There was a significant main effect on RTs for *numerical magnitude* (*F*(3, 48) = 24.281, *p*_*GG*_ < .001, ε_GG_ = .712) and *notation* (*F*(2, 32) = 72.226, *p*_*GG*_ < .001, ε_GG_ = .803). There was no main effect of *side* (*F*(1, 16) = .496, *p* = .491), but a significant interaction *side* * *numerical magnitude* (*F*(3, 48) = 14.076, *p*_*GG*_ < .001, ε_GG_ = .598), indicating a possible SNARC effect. We also observed a significant *notation* * *numerical magnitude* interaction (*F*(6, 96) = 11.556, *p*_*GG*_ < .001, ε_GG_ = .564), as larger numbers were processed slower in all notations, but especially so in *Chinese hand signs* and *Chinese characters*, which may reflect the fact that visual complexity increases in these notations. Crucially, the three-way *notation* * *numerical magnitude* * *side* interaction that would have indicated a different SNARC effect depending on the notation was not significant (*F*(6, 126) = 1.427, *p*_*GG*_ = .232, ε_GG_ = .716), and no main effect (*F*(5, 16) = 0.515, *p* = .761) of factor *order* was significant, with the only significant interaction being *order* * *side* (*F*(5, 16) = 6.498, *p* = .002; all other interactions n.s., *p* > .13), indicating that participants were faster in the mapping they had learned first. Left-handed and right-handed RTs from this task are plotted in Fig 2.

We then computed dRTs for each number and used these to run regressions of dRTs by number, see Table 1. The resulting slopes were tested for difference against 0 to investigate if a SNARC effect existed in each notation, as negative slopes would indicate smaller dRTs (i.e., relatively faster right-handed responses) the larger the number. They revealed a SNARC effect for *Arabic digits* (*t*(21) = -5.461, *p* < .001), *Chinese characters* (*t*(21 = -3.395, *p* = .003), and for *Chinese hand signs* (*t*(21) = -2.879, *p* = .009. BFs were calculated following the logic proposed by Dienes [49,50], comparing the likelihood of a point-hypothesis at 0 to that of a uniform distribution ranging from 0 to the dRT slope reported in a meta-analysis by Schiller et al. [54] of -5.74 ms/digit. They confirm that the effect in *Arabic digits* (BF > 1000; decisive evidence), as well as both *Chinese characters* (BF = 147.6; decisive evidence) and *Chinese hand signs* (BF = 30.2 strong evidence) are robust effects. Bayes factors testing for a real difference between dRTs obtained from our experiment by comparing likelihoods of a point-0 H0 to an H1 of slope(dRT_notation1_) = slope(dRT_notation2_) reported strong or decisive evidence for the H1 in all cases (all BFs > 10), indicating that dRT slopes were comparable and the non-significant three-way interaction indeed pointed towards no difference. Testing for a MARC-like effect of an advantage for left-odd and right-even responses revealed no evidence of such an effect, as dRTs did not differ between odd and even numbers (*t*(21) = -0.799, *p* = .433). For comparability with other studies, we calculated slopes for both dRT by number and dRT by magnitude bin, see Table 1.

### 2.6 Discussion

Our results from a parity judgement task indicate that there was a clear horizontal SNARC effect in all notations. Importantly, and unlike previous results [20], this includes *Chinese characters*. This could be explained by differences in reading habit between participants from Taiwan [20] and mainland China (this study). But it should also be noted that this is the first time that a SNARC effect could be demonstrated in *Chinese hand signs*. The slope of -5.1 ms/digit for *Arabic digits* compared well to a recent meta-analysis of SNARC parity judgement experiments found in Western participants (-5.74 ms/digit [54]). The effect was not significantly smaller in other notations (all *p* > .06, which according to our Bayes factors was not just a product of insensitive data, although the explained variance varied somewhat, see Table 1. This is supported by the fact that BFs gave strong evidence in favour of a horizontal SNARC effect in all notations.

## 3 Experiment 2: Magnitude judgement

Experiment 2 used the same procedure as experiment 1, with the main difference that we used a magnitude judgement paradigm. That is, participants judged whether a given number was smaller or larger than 5. We chose to conduct another experiment since some participants told us during experiment 1 that they used visual features of some of these notations to complete the task. Thus, we used a different task with a different grouping of numbers, and additionally presented participants with a self-designed questionnaire following the experiment in which we asked them about any strategies used, allowing us to test if any SNARC effect we would find would be robust to such strategies that might not rely on processing numerical magnitude (i.e., the main driver of the SNARC effect).Since the stimulus features would still allow completing the magnitude judgement task in Chinese hand signs based primarily on visuospatial features (see Fig 1), and it has been suggested that semantic processing of Chinese characters and Chinese hand signs may occur slightly later in processing than for Arabic digits [35], we also included analyses of numerical distance effects. A numerical distance effect suggests semantic processing of the stimuli [7,42], since it can best be explained by numerical magnitude, which is to say the meaning of a numerical stimulus.

### 3.1 Participants

Twenty-five native Chinese speakers (at least 18 years old, age: M = 24.4, SD = 2.6), recruited September and October 2013, participated voluntarily for an agreed pay of 30 RMB. All were right-handed by self-report, 11 were female. All had normal or corrected-to-normal vision. All were university students, 18 years or older, either enrolled or doing project work at Tsinghua University, Beijing and naive to the purpose of the task. Written, informed consent was obtained prior to the experiment from each participant according to the 1964 Declaration of Helsinki. Data were kept anonymously and could not be linked to names on the consent sheets–see section 2.1 for details.

### 3.2 Stimuli and apparatus

Participants were seated approximately 60 cm in front of an Acer (Acer Inc., New Taipei, Taiwan) laptop computer with a 15.6” flat screen (effective screen diagonal: 40 cm) running at 1024 * 768 pixel with a refresh rate of 60 Hz. Responses were given via an integrated German QWERTZ-keyboard. The stimuli used were identical to those in experiment 1. Due to the different screen, the size was slightly different at app. 20 mm * 20 mm, or approximately 2 degrees of visual angle. The main difference to experiment 1 was that participants now had to decide whether a given number was smaller or larger than 5 (magnitude judgement task). After testing, we administered a self-designed 9-item questionnaire containing mainly multiple-choice questions about their perception of the experiment and, crucially, about whether or not they used strategies other than number processing in any of the conditions. The question on the use of strategies was split between one multiple-choice item asking whether or not strategies were used, and an open-ended question asking participants who answered “yes” what strategies they used.

### 3.3 Procedure

The experiment was segmented into 6 blocks: Two blocks (*left = small* and *left = large*) for each of the three notations, *Arabic digits*, *Chinese characters*, and *Chinese hand signs*. The blocks, groups, instructions and feedback were analogous to experiment 1. Following the experiment, participants filled out the questionnaire. In total, the experiment lasted between 50 and 65 minutes.

### 3.4 Data analysis

One participant had to be excluded from analysis due to the number of errors made (more than 2 SD above the mean), leaving 24 participants for analysis. The data were analysed in the same way as in experiment 1 by computing an ANOVA, followed by dRTs and slope analyses over trimmed RTs, see section 2.4 for details. We also computed BFs for comparing a point-hypothesis at 0 to a uniform distribution from 0 to the mean dRT slope for magnitude judgement experiments (-7.9 ms/digit) reported in a recent meta-analysis [54], see section 2.5. 1.7% of trials were excluded through the trimming procedure. To test for distance effects, we ran another ANOVA with the factors *notation* (3 levels) and *distance* from comparison (i.e., |x-5|, 4 levels) over response times, followed by regression analyses of dRT by number for each notation, see section 2.4.

### 3.5 Results

Again, we found a significant main effect for *numerical magnitude* (*F*(3, 54) = 56.607, *p*_*GG*_ < .001, ε_GG_ = .761) and *notation* (*F*(2, 36) = 88.489, *p*_*GG*_ < .001, ε_GG_ = .932), and this time also for *side* (*F*(1, 18) = 8.159, *p* = .010) on RTs. There were also significant interactions *side* * *magnitude* (*F*(3, 54) = 9.586, *p*_*GG*_ < .001, ε_GG_ = .521) and *notation* * *magnitude* (*F*(6, 108) = 15.022, *p*_*GG*_ < .001, ε_GG_ = .774), the former indicating a SNARC effect. Again, no three-way interaction of *side* * *magnitude* * *notation* was observed (*F*(6, 108) = 1.973, *p*_*GG*_ = .130, ε_GG_ = .491). Similar to Experiment 1, the order of the tasks had hardly any effect on these results: There was no significant main effect of the factor order (*F*(5, 18) = 0.597, *p* = .703) and only the 3-way interaction of the factors *order* * *side* * *magnitude* (*F*(15, 54) = 2.681, ε_GG_ = .521, *p*_*GG*_ = .026) was significant. All six other interactions with factor *order* were not significant (all *p* > .05). Future research and replications will need to clarify whether this significant 3-way interaction indicates a modulation of the SNARC effect by order of blocks, or whether this is a false positive due to testing of multiple interactions [55].

As in experiment 1, we computed regressions of dRTs by number (see Table 1). There was a significant SNARC effect for *Arabic digits* (*t*(23) = -4.770, *p* < .001), a trend for *Chinese characters* (*t*(23) = -1.958, *p* = .062), and no significant SNARC effect for *Chinese hand signs* (*t*(23) = -0.943, *p* = .356). Similarly, our Bayesian analysis shows decisive evidence for a SNARC effect in *Arabic digits* (BF > 1000), substantial evidence for a SNARC effect in *Chinese characters* (BF = 4.5), but inconclusive data regarding the effect in *Chinese hand signs* (BF = 1.1). RTs for left-handed and right-handed responses from this task can be seen in. Bayes factors testing for differences between dRTs in the data from this experiment were much less clear in experiment 2, giving substantial evidence for the notations *Arabic digits* and *Chinese characters* being similar (BF = 3.1), but showed that the data were in fact insensitive to detect a difference or absence thereof on the other comparisons (*Arabic digits* vs. *Chinese hand signs*: BF = 0.4; *Chinese characters* vs. *hand signs*: BF = 1.1).

An ANOVA with factors *notation* and *distance* over RTs revealed main effects of *distance* (*F*(3, 69) = 93.289, *p*_*GG*_ < .001, ε_GG_ = .828), *notation* (*F*(2, 46) = 71.528, *p*_*GG*_ < .001, ε_GG_ = .990), as well as a *notation* * *distance* interaction (*F*(6, 138) = 4.001, *p*_*GG*_ = .003, ε_GG_ = .751). Regression slopes of distance by RT were significant for all notations (Arabic digits: *t*(23) = -6.823, *p* < .001; Chinese characters: *t*(23) = -16.218, *p* < .001; Chinese hand signs: *t*(23) = -5.674, *p* < .001).

**Fig 3:**
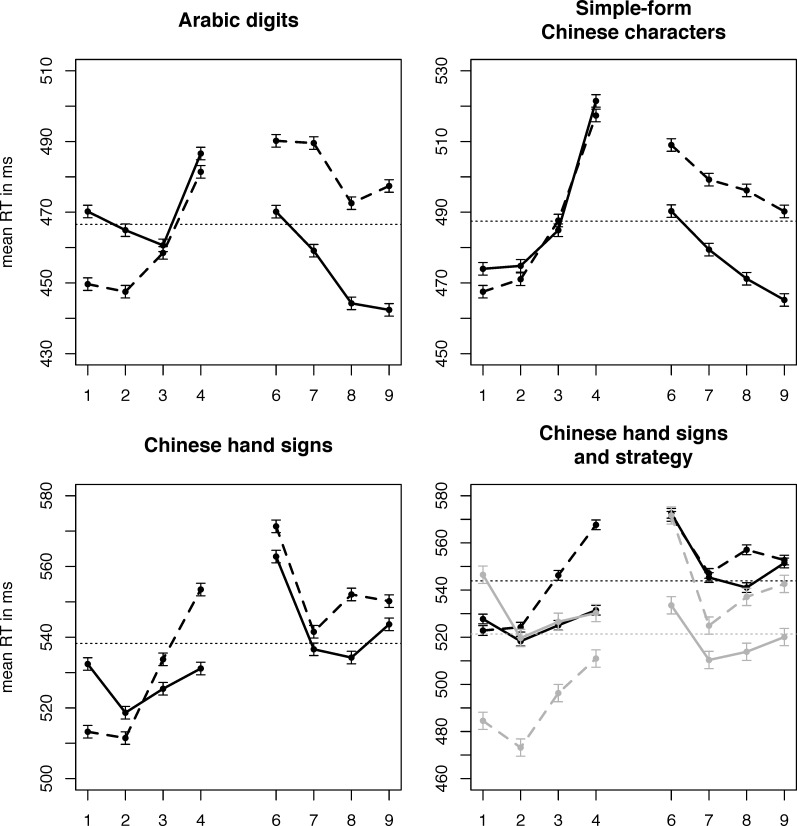
Left-handed and right-handed responses to each number in each notation, magnitude judgement. **SNARC effect**: Right-handed responses slower than left-handed responses for small numbers, faster for large numbers. **Distance effect**: Increased responses times for numbers closer to the middle. Bottom right: Participants who reported using visual categorisation (per our questionnaire; plotted in grey) vs. those who did not. Note the slightly compressed y-axis in this plot. Error bars indicate within-subject SEMs for each number, pooled across each contrast of numbers [52,53]. Horizontal dashed lines indicate grand means of RTs for each notation.

Asking participants about their use of strategies other than number processing (see section 3.1 for the motivation, 3.2 for details on the questionnaire) revealed that 8 of 24 participants had used some visual strategy. One strategy was reported by multiple participants: 6 participants stated having categorised the shape of hand gestures by visual features, such as complexity or “straightness” of fingers to decide if numbers were smaller or larger than 5. We re-analysed the notation *Chinese hand signs* separately for participants who used this visual strategy and for those who did not. Grouping participants like this gave us mean regression coefficients of *dRT* by *number* of y = 65.3–11.6x for participants who reported having used categorization based on visual features, and y = -11.3 + 0.4x for participants who did not. Both of these slopes were not significantly different from 0 (both *p* > .19). However, the Bayesian analysis revealed substantial evidence for the effect being truly non-existent in participants not using a strategy (BF = 0.3), while the data were insensitive for participants using a visual categorisation strategy (BF = 1.9). Interestingly, the distance effect was present for both groups (visual strategy: -4.3 ms/digit; no visual strategy: -7.2 ms/digit), see Fig 3.

### 3.6 Comparing the experiments

Running a 3 (*notation*) * 2 (*side*) * 4 (*numerical magnitude*) * 2 (*experiment*) ANOVA over RTs revealed a significant main effect for experiment. Participants responded faster in magnitude judgement (*F*(1, 44) = 6.655, *p* = .013). This was the case for all notations, as supported by the fact that there was no *experiment* * *notation* interaction (*F*(2, 88) = 0.495, *p*_*GG*_ = .609, ε_GG_ = .987). However, there was no interaction of either *side* * *numerical magnitude* * *experiment* (which would indicate a different SNARC effect for each experiment–*F*(3, 132) = 0.460, *p*_*GG*_ = .577, ε_GG_ = .499) or *side* * *numerical magnitude* * *notation* * *experiment* (which would indicate that notational differences in the SNARC effect depend on the type of experiment–*F*(6, 264) = 0.442, *p*_*GG*_ = .748, ε_GG_ = .568).

**Fig 4:**
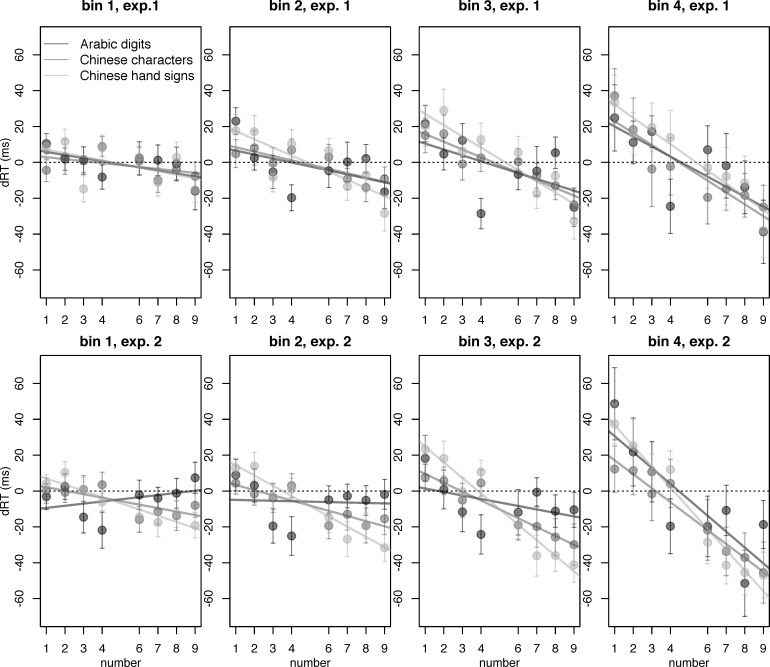
dRTs computed from vincentized RTs. Colours indicate the three notations, Arabic digits, Chinese characters, and Chinese hand signs. Bins in ascending order by RT (i.e., bin 1 contains the fastest responses). Note the more strongly negative dRT slope in bins with slower RTs.

As it has also been suggested that space-number associations become more salient the longer a participant takes to react [56], we conducted an analysis in which we aggregated RTs by latency bins. For this, we vincentized the data, such that for each quantile of the RT-distribution, separate means were computed [57,58]. We applied this procedure by calculating in each experiment vincentized RTs for each experimental condition (i.e., 2 side x 2 number x 3 notation). These vincentized RT were then used to calculate dRTs and the corresponding dRT slopes in the usual fashion (Fig 4). As expected [56], in both experiments and for all notations, dRT slopes decreased by bin (i.e., became more strongly negative, indicating a stronger SNARC effect). A 2 (*experiment*) * 3 (*notation*) * 4 (*bin*) ANOVA over dRT slopes revealed that this effect was statistically significant (main effect of *bin*: *F*(2, 88) = 29.177, *p*_*GG*_ < .001, ε_GG_ = .557), in a way that could not be explained by any interaction with another factor (interaction *bin* * *notation*: *F*(4, 176) = 2.365, *p*_*GG*_ = .087, ε_GG_ = .613; interaction *bin* * *experiment*: *F*(2, 88) = 0.454, *p*_*GG*_ = .524, ε_GG_ = .557; all other interactions also n.s., all *p* > .3).

### 3.7 Discussion

In the magnitude judgement task, our regression analysis showed a clear horizontal SNARC effect in *Arabic digits* (-7.5 ms/digit; again very similar to the -7.90 ms/digit found for magnitude judgement by a recent meta-analysis [54]) a trend towards a SNARC effect *in Chinese characters*, and no significant SNARC effect in *Chinese hand signs*. Still, while the t-tests failed to reach significance, the ANOVA revealed no significant interaction between the SNARC effect and notation, indicating that these data do not support the non-existence of a horizontal SNARC effect. Bayes factors revealed that our data were too noisy to take this as strong evidence either for or against differences in the strength of the SNARC effect. In fact, even t-tests comparing regression slopes over dRT directly did not provide evidence for a difference in effects (all *p* >.10), and Table 1 shows the slopes to be quite similar. This is consistent with the Bayesian results, which indicate that there was a horizontal SNARC effect in *Chinese characters*, and the data were insensitive in the case of *Chinese hand signs*, rather than there being no effect.

## 4 General Discussion

For the first time, we demonstrated a horizontal SNARC effect in *Chinese characters* and *Chinese hand signs*. The only other study known to us that used Chinese characters was conducted in Taiwan, and no horizontal SNARC effect was found [20]. However, participants in this study were assumed to have different experiences of reading and writing in Chinese from our participants in Mainland China. Our results thus indicate that not finding a SNARC effect for Chinese characters in Taiwanese participants cannot be attributed to the notation alone. We also found a horizontal SNARC effect for Chinese hand signs, consistent with the idea that the spatial mapping of numbers is independent of notation and an integral part of number processing [59]. At the same time, it is worth mentioning that some previous studies have proposed a qualitatively different processing of Chinese characters and by Chinese speakers in general [35,36], in which case it is by no means obvious to expect similar effects. The SNARC effect was also less robust in Chinese hand signs than in other notations. Our data allow us only to speculate why this might be the case, but it is possible that this was due to a confounding influence of visual features like finger numerosity, which has been proposed to evoke space-number association [18] and could have interfered with a SNARC effect of number magnitude. Indeed, participants responded faster with the right hand to hand signs for 3 and 4 (represented through relatively high numerosities within our stimulus range). Still, the overall result is that the SNARC effect persists in Chinese characters and hand signs, with minor differences depending on experiment or notation. This emphasises that it may be a promising tool to further investigate the mechanisms involved in the processing of Chinese numerals, and of numbers by Chinese speakers regardless of notation. Further research may also investigate which resources are used in the processing of these different notations. Finding a SNARC effect in Chinese hand signs makes this notation a potential tool to investigate phenomena of embodied numerical cognition. Our experiments do not allow such tests, as we did not manipulate any features of the hand signs, but there are some predictions of the embodiment theory that could be tested [31]: This theory predicts that finger-counting habits would impact the association of space and number, as should the orientation or posture of the hands. Finally, hand signs above 5 also offer the possibility of testing numerical cognition in hand signs that are not defined by finger numerosities, thus separating embodiment and numerosity.

Considering that the mechanisms giving rise to the SNARC effect likely differ somewhat between magnitude judgement and parity judgement [3,41], it was not clear whether to expect a similar effect in both our experiments. Our analyses revealed no quantifiable effect of the task on the SNARC effect, indicating that task differences may not have a big influence on the size of the effect. However, other properties of the data reflect the differences between the two tasks, as we found that there was vastly more inter-individual variability in the magnitude judgement task, which also led to the fact that SNARC effects in Chinese hand signs and Chinese characters failed to reach significance in magnitude judgement but not in parity judgement. We also observed the typical shape of the SNARC effect with almost constant dRTs on each side of the standard, but a big offset between the two sides (compare the distance between lines in Fig 3 to e.g. Fig 2 of [56]). These dRTs, along with dRTs split by latency quantile, can be seen in Fig 4. We split responses into four quantiles by latency to test for the time course of the SNARC effect in our experiment, as it has been proposed that the magnitude of the effect may increase for slower responses [56]. Splitting responses into bins by latency following the vincentizing procedure proposed by Ratcliff [57], we observed that in both experiments, slower responses showed a markedly stronger SNARC effect. This is consistent with previous results [56] and the time course of several other similar effects [60]. Gevers et al. [56] suggested that this may be due to weak activations taking longer to take effect, so that they would not influence relatively fast responses, which would in consequence show an on average weaker SNARC effect than slower responses. Our experiments did not test this prediction, but our results are very much in line with it.

It may be interesting in future studies to use these two Chinese notations to study the underlying mechanisms of the SNARC effect. Our finding of a robust distance effect across all participants, notations and number ranges in magnitude judgement represents evidence that numerals were indeed processed semantically in all these conditions, compatible with Liu et al.’s [36] view that Chinese numerals are encoded in parallel in multiple modalities. Of course, it is not surprising for Chinese native speakers to automatically process the meaning of hand signs–what is interesting is that processing to this level was fast enough to be detected in our task, as evidenced by the strong numerical distance effect.

This distance effect was present even when participants employed a visual categorisation strategy, and there was no significant speed difference between the two groups (*p* = .524, mean RT without strategy: 544 ms, with strategy: 521 ms). Indeed, this may serve as one possible explanation why somewhat counter intuitively and contrary to what we would have predicted, the SNARC effect was notably stronger instead of weaker in participants using a categorisation strategy vs. those who did not. If semantic processing occurred even when participants employed visual categorization, then it is not surprising to find the usual SNARC effect, in addition to a possible amplification of the typical offset [56] between two sides of a standard in magnitude judgement. In fact, the difference between these two groups was so large that participants who did use a strategy displayed the largest SNARC effect we found in our experiment, while the participants who did not showed no SNARC effect at all. This is certainly compatible with the idea that in each task, different mechanisms beyond semantic processing (verbal in parity judgement, visuospatial in magnitude judgement) contribute to the effect, although the small number of participants reporting categorisation gives us only rather noisy data on this.

## 5 Conclusion

We found a horizontal SNARC effect in all three notations. For Chinese characters and Chinese hand signs, this is the first time that we know of that a horizontal SNARC effect has been demonstrated. These effects were slightly smaller than in Arabic digits. The effect persisted in all notations in the parity judgement task, with more mixed results in magnitude judgement. This speaks for the ubiquity of the SNARC effect in number representation and can be used in further research on differences in number processing between Chinese speakers and Western participants.
